# Therapeutic Pearls at Random Strung

**Published:** 1881-01

**Authors:** 


### THERAPEUTIC PEARLS AT RANDOM STRUNG.

(Under this heading will be published alphabetically, as far as practicable, such notes and formula, from various sources, as are likely to prove serviceable to the busy practitioner. Many of them have been copied for years, and the sources from which they were obtained have been forgotten, as they were intended originally for private reference or use.)

### AMMENORRHŒA, DYSMENORRHŒA, &C.

Dr. W. K. Bowling, of Nashville, Tenn., regards spirits of ammonia as specific in all the above diseases, in relieving the terrible sufferings of the patient. Use the following, viz: Put a drachm of spirits of ammonia in a chamber, and require the patient to sit on it a few moments. Dr. Bowling regards this a "Discovery" in medicine.

### ASTHMA.

℞ Chloral Hydrate, - - - - - - ʒ v.

Potassii Bromidi, - - - - - ʒ ijss.

Syrup Flores Aurantii, Aquæ Distillat, - aaf ℥ i.

M.

S. One teaspoonful in half a wine glassful of water every two hours until sleep is induced or dysponœa is relieved.

### ASTHMA.

℞ Grindelia Robusta, - - - - - ¾ ounce.

Yerba Santa, - - - - - - - ¼ ounce.

M. Sig.: One-half to one teaspoonful, more or less, according to age, every half, one or two hours; be governed by its effect on the disease.

Milan, Mo. J. C. WEBB, M. D.

### ASCARIDES.

℞ Epsom Salts, - - - - - 2 ounces.

Common Salt, - - - - - ½ ounce.

M. Give two tablespoonfuls in half glass water, with five drops carbolic acid, night and morning.

The salt and carbolic acid will destroy the worms, and the brisk action of the salts will carry them out. Persevere.

Caseyville, Ky. D. M. BARKLEY, M. D.

### ACNE.

Red Lotion, composed of two scruples of bichloride of mercury, one of the bisulphuret and ten minims of creosote in a pint of water is to be used twice a day.

### ALBUMENUREA AND DROPSY.

Steep the inner bark of the common elder (Sambucus Canadensis), in hard cider, and give in ounce doses three or four times a day.

### AMENORRHŒA.

This and dysmenorrhœa are said to be successfully treated by quinine, applied locally to the lower two-thirds of the spine, after having irritated the surface with mustard and alcohol.

ADONIS VERNALIS.—This drug has been shown by a series of experiments, published in the *St. Petersburger Medical Wock.* (Jan. 6, 1879), to be analagous in its action to digitalis, strongly affecting the heart, but having certain peculiar properties, one of them being the total absence of dangerous effects, even when it has been taken for a long time continuously.

INF. ADONIDIS VERNALIS CONC. 1-7. Dose—½ to 1 fluid ounce.

ALBUMINATE OF IRON.—This is a solution of hydrate of iron in combination with egg albumen, and it is said to be more easily assimilated than any other prepariation of iron. It is used in chlorosis, and in all cases of an anæmic character. In rachitis it should be associated with a minute proportion of phosporus, and in all cases should be freshly prepared. The strength is about half a grain of hydrate of iron in a fluid drachm.

AMYL NITRITE (*purified*).—Sir J. Y. Simpson found the inhalation of two drops of this body to give immediate relief in cases of nervous cephalalgia, however severe. The experience of Dr. Douglas Lithgow is identical with this. When blueness of the face and stertorous breathing come on after the administration of chloroform, a few drops of nitrite of amyl held to the patient's nose, on lint, almost invariably effects restoration of the breathing and color, and rapidly brings on sickness and vomiting (C. Bader.) Berger has used it with success in two cases of acute cerebral anæmia with severe fainting. The preparation must be pure and without acid reaction. The dose usually given by him was two, five, or ten drops. Dr. Lauder Brunton testifies to the immediate relief afforded in *angina pectoris* by the inhalation of 5 to 10 drops of the pure nitrite. Mr. Lennox Brown says "Five measured minims of a mixture of equal parts nitrite of amyl and rectified spirits may be safely given. . . . . . The effect of nitrite of amly as of aldehyde, ether, or chloroform, is much more powerful if added to hot water and inhaled with steam." Nitrite of amyl is also said to be of use in sea-sickness. *Unless otherwise directed, we always dispense and supply* AMYL NITRITE *as purified by ourselves.* The safest and most convenient general way of perscribing and using this powerful remedy is in the form of small glass CAPSULES containing two or three minims; one of these is broken in the corner of a handkerchief and held to the nose.

ANTHROBOMA.—This preparation has for its basis a selected part of the fruit of *Theobroma Cacao.* Though specially serviceable in those forms of dyspepsia which are accompanied by intolerance of food, it forms an agreeable and refreashing dietetic beverage for general use, and can be enjoyed by the most delicate.

APIOL.–Apiol is a nearly colorless non-volatile liquid, insoluble in water, very soluble in alcohol, ether, and chloroform. The alcoholic solution reddens litmus. Apiol is colored by sulphuric aicd. It has been recommended as a powerful febrifuge; it may be administered in doses of five to six drops diffused through any bland liquid or preferably in the form of PERLES (*q. v.*) In cases of intermittent fever, seven to fifteen grains may be given daily in one dose five or six hours before the paroxysm. It may also be given in the form of a syrup.

APOMORPHIA HYDROCHLORIDE. — Apomorphia is an artificial base derived from morphia some few years ago by Drs. Matthiessen and Wright. It differs from morphia very considerably in its physiological effects. One-tenth of a grain of the hydrochloride injected subcutaneously, or one-fourth administered internally, produces vomiting in four to ten minutes. Dr. Gee finds that it is a powerful anti-stimulant and a non-irritant emetic. It has been recommended as a remedy for sea-sickness, in doses of 1-60 to 1-40 of a grain, and in similar or slightly larger doses Dr. Jurasy has employed it with gratifying results as an expectorant in bronchitis and tracheitis.

LIQUOR APOMORPHIÆ HYDROCHLOR.—Dose: One fluid drachm.

AROMATIC TETRACHLORIDE OF CARBON.—Tetrachloride of Carbon was brought into notice as an anæthetic and sedative by Dr. Protheroe Smith, in a series of papers which appeared in the *Lancet* of May and June, 1867. It is a colorless, exceedingly volatile liquid, having a delicate odor not unlike that of quince. From Dr. Protheroe Smith's papers it appears that when inhaled, this body is very effective in removing the pains of labor and of dysmenorrhœa, and that it has been extensively and successfully employed for headache, toothache, neuralgia, lumbago, and rheumatic pains, applied externally on American leather cloth. Dr. Protheroe Smith has found it very efficacious in the mitigation and cure of hay-fever. He usually prescribes a very purified and perfumed compound manufactured by CORBYN & COMPANY which he calls *Aromatic Tetrachloride of Carbon*, and for which there is considerable demand. "This new sedative, to which Dr. Protheroe Smith called attention in the *Lancet* about ten years ago, is prepared in a very agreeable form by Messrs. CORBYN. It has an odor like that of quince, and is said to have been useful in removing the pains of labor and dysmenorrhœa, in hay-fever and in neuralgia."—*Lancet*, July 13, 1878.

